# Au- ZnFe_2_O_4_ hollow microspheres based gas sensor for detecting the mustard gas simulant 2-chloroethyl ethyl sulfide

**DOI:** 10.1007/s44211-024-00573-z

**Published:** 2024-04-30

**Authors:** Junchao Yang, Molin Qin, Yong Pan, Liu Yang, Jianan Wei, CanCan Yan, Genwei Zhang, Shuya Cao, Qibin Huang

**Affiliations:** State Key Laboratory of NBC Protection for Civilian, Beijing, 100000 China

**Keywords:** 2-CEES, Au loading, Chemical warfare agent, Gas sensor, ZnFe_2_O_4_

## Abstract

Mustard gas, a representative of blister agents, poses a severe threat to human health. Although the structure of 2-chloroethyl ethyl sulfide (2-CEES) is similar to mustard gas, 2-CEES is non-toxic, rendering it a commonly employed simulant in related research. ZnFe_2_O_4_-based semiconductor gas sensors exhibit numerous advantages, including structural stability, high sensitivities, and easy miniaturization. However, they exhibit insufficient sensitivity at low concentrations and require high operating temperatures. Owing to the effect of electronic and chemical sensitization, the gas-sensing performance of a sensor may be remarkably enhanced via the sensitization method of noble metal loading. In this study, based on the morphologies of ZnFe_2_O_4_ hollow microspheres, a solvothermal method was adopted to realize different levels of Au loading. Toward 1 ppm of 2-CEES, the gas sensor based on 2 wt.% Au-loaded ZnFe_2_O_4_ hollow microspheres exhibited a response sensitivity twice that of the gas sensor based on pure ZnFe_2_O_4_; furthermore, the response/recovery times decreased. Additionally, the sensor displayed excellent linear response to low concentrations of 2-CEES, outstanding selectivity in the presence of several common volatile organic compounds, and good repeatability, as well as long-term stability. The Au-loaded ZnFe_2_O_4_-based sensor has considerable potential for use in detecting toxic chemical agents and their simulants.

**Graphical abstract:**

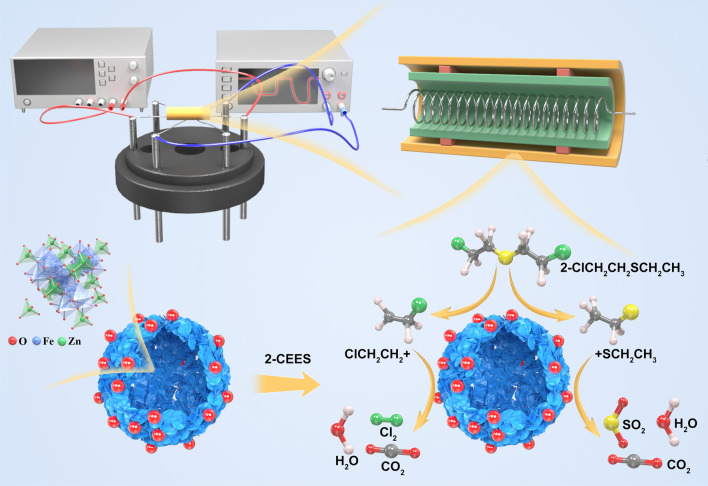

## Introduction

Mustard gas, as a representative toxic chemical vesicant, has been extensively used in chemical warfare. It is the most produced and stored chemical weapon to date, causing significant casualties and posing a grave threat to human health and societal stability. Mustard gas is also regarded as the “king of the toxins,” [1–4] and exhibits high toxicity, with an “immediately dangerous to life or health” concentration of 0.1 ppm [5]. Notably, no antidotes for mustard gas have been reported thus far, and only symptomatic treatments can be administered. Therefore, the rapid, accurate detection and identification of mustard gas are critical [6, 7]. The non-toxic simulant 2-chloroethyl ethyl sulfide (2-CEES) has a chemical structure similar to that of mustard gas; therefore, it is commonly used in related research [8–10].

Currently, the primary methods of detecting toxic chemical agents and their simulants include laser [11], infrared [12], Raman [13], and ion mobility spectroscopies [14], mass spectrometry [15], surface acoustic wave sensing [16], and gas sensing using metal oxide semiconductors [17–19]. Owing to their simplicity, low costs, and ease of miniaturization [20–22], metal oxide semiconductor sensors have received considerable attention, and have been employed in detecting toxic gases at low concentrations. In addition, sensing materials, such as WO_3_ [23], ZnO [24], CdSnO_3_ [25], and Fe_2_O_3_ [26], have been applied in detecting the mustard gas simulant 2-CEES.

The binary metal oxide ZnFe_2_O_4_ is characterized by a narrow bandgap (2.1 eV), structural stability, and abundant oxygen vacancies. It has recently attracted attention in the field of gas sensing [27–31]. In a previous study [17], a ZnFe_2_O_4_ microsphere-based gas sensor was designed for detecting 2-CEES; however, challenges such as high operating temperatures, inadequate sensitivity at low concentrations, and prolonged response/recovery times still need to be addressed. Numerous studies have reported that the gas-sensing performance of metal oxide sensors can be significantly enhanced through noble metal loading [32–35], heterogeneous ion doping [36–38], and heterojunction formation [39–41]. Notably, noble metals with catalytic properties, such as Au, Ag, Pt, and Pd, may enhance the reaction efficiencies of target gas molecules on the surfaces of semiconductor metal oxides and reduce the activation energy required for the reaction. Accordingly, the sensitivities of gas sensors and their response/recovery rates may be improved while reducing their levels of energy consumption. This mechanism of sensitivity enhancement is known as the effect of chemical and electronic sensitization caused by noble metals [42–47]. As an example, Kim et al. prepared a 0.6 wt.% Pd-loaded ZnO nanofiber-based gas sensor with a sensitivity of 74.7 toward 100 ppb H_2_ at a working temperature of 350 °C [48]. Patil et al. fabricated a Pt-CdSnO_3_ thin-film sensor, and compared to the pure CdSnO_3_ thin film, it exhibited a superior response sensitivity toward 2-CEES as well as enhanced response and recovery times [49].

In this study, Au-loaded ZnFe_2_O_4_ hollow microspheres were prepared using a solvothermal method and used to fabricate gas sensors. Gas sensitivity studies were performed using the mustard gas simulant 2-CEES, and the enhanced sensing mechanism was revealed via microstructural characterization.

## Material and methods

### Materials and reagents

The chemicals used in this study, including Zn(NO_3_)_2_·6H_2_O, Fe(NO_3_)_3_·9H_2_O, urea, glycerol, isopropanol, ethanol, HAuCl_4_·4H_2_O, and sodium citrate dihydrate (with a relative molar mass of 258.07 g/mol), were obtained from Sinopharm (Beijing, China). The reagents were of analytical grade and used without further purification.

### Synthesis and preparation of the materials

Firstly, 0.252 g of HAuCl_4_·4H_2_O and 0.237 g of sodium citrate dihydrate were separately dissolved in deionized H_2_O in 10 mL volumetric flasks, and and the solutions were then diluted to the mark.

Secondly, Zn(NO_3_)_2_·6H_2_O (0.5 mmol, 0.149 g), Fe(NO_3_)_3_·9H_2_O (1.0 mmol, 0.404 g), and urea (1 mmol, 0.060 g) were added to the sodium citrate solution, along with 0, 100, 200, 300, or 400 µL of the HAuCl_4_ solution. The resulting solution was added to a mixture of glycerol and isopropanol (4 and 16 mL, respectively), which was then stirred for 30 min. The solution was transferred to a 50 mL reaction vessel, and the hydrothermal reaction was allowed to proceed at 180 °C for 24 h. Subsequently, the solution was centrifuged in deionized H_2_O and then ethanol at 9000 rpm for 8 min, with the centrifugation protocol repeated three times. The solution was then dried in a vacuum oven at 80 °C for 12 h, and finally, the dried sample was calcined in a muffle furnace at 450 °C, with a heating rate of 2 °C/min, for 2 h. Thus, ZnFe_2_O_4_ samples loaded with 0, 1, 2, 3, or 4 wt.% Au were obtained.

### Characterization of the sensing materials

The structural morphology was observed using scanning electron microscopy (SEM, Gemini 300, ZEISS, Oberkochen, Germany) and transmission electron microscopy (TEM, JEM-F200, JEOL, Tokyo, Japan). The crystalline phases were determined via X-ray diffraction (XRD, Empyrean, Malvern Panalytical, Malvern, UK) with a Cu target and Kα1 radiation (λ = 0.154056 nm). The elemental compositions were analyzed using energy-dispersive X-ray spectroscopy (EDS, JED-2300T, JEOL). The chemical compositions were determined using X-ray photoelectron spectroscopy (XPS, K-Alpha, Thermo Fisher Scientific, Waltham, MA, USA) at a working voltage and filament current of 12 V and 6 mA, respectively. The working voltage and filament current of 12 V and 6 mA, respectively. The electron binding energies were calibrated using the C 1s peak (284.8 eV).

### Fabrication and evaluation of the gas-sensing device

**Fig. 1:**
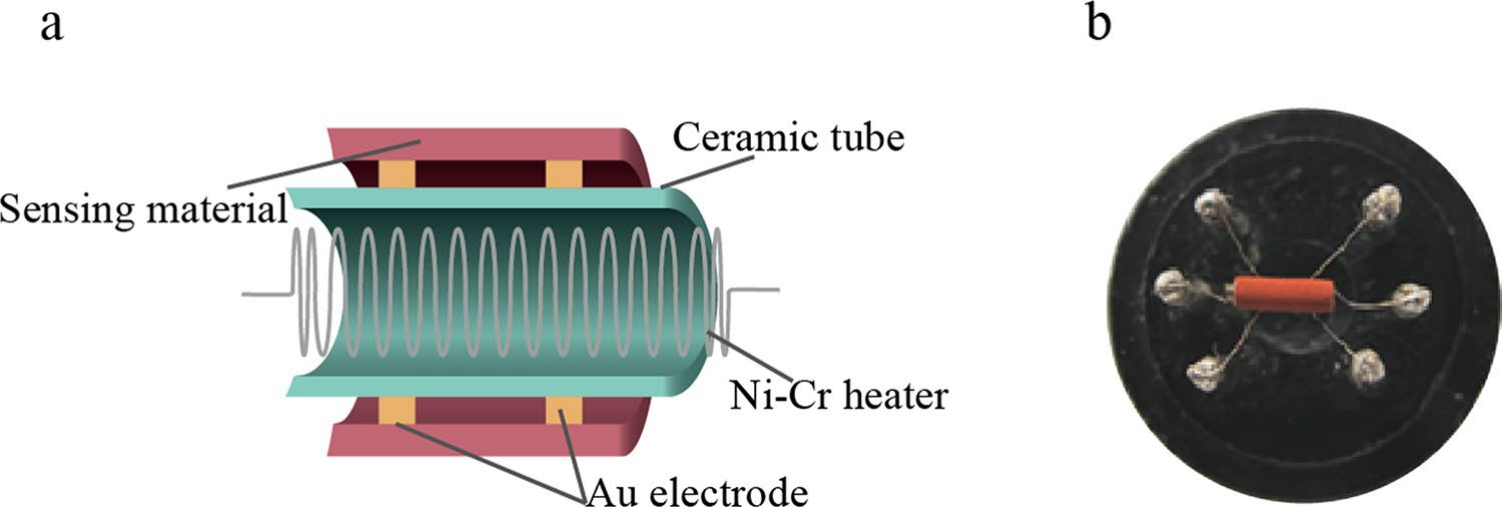
Schematic of the gas-sensing device: **a** Structure of the side-heated sensor and **b** image of the real product [17]

The gas sensor was fabricated as follows. First, the as-prepared ZnFe_2_O_4_ sample was added to a suitable amount of deionized water H_2_O to form a uniform slurry. The slurry was evenly coated onto ceramic Al_2_O_3_ tubes (with Au electrodes at both ends and Pt wires as leads for the testing electrodes) using a fine brush to form thin films. After drying in the air, the samples were sintered at 300 °C for 2 h. Subsequently, a Ni–Cr alloy heating wire was placed inside the ceramic tube and welded to a six-legged base; the procedure is shown in Fig. 1a, b.

**Fig. 2:**
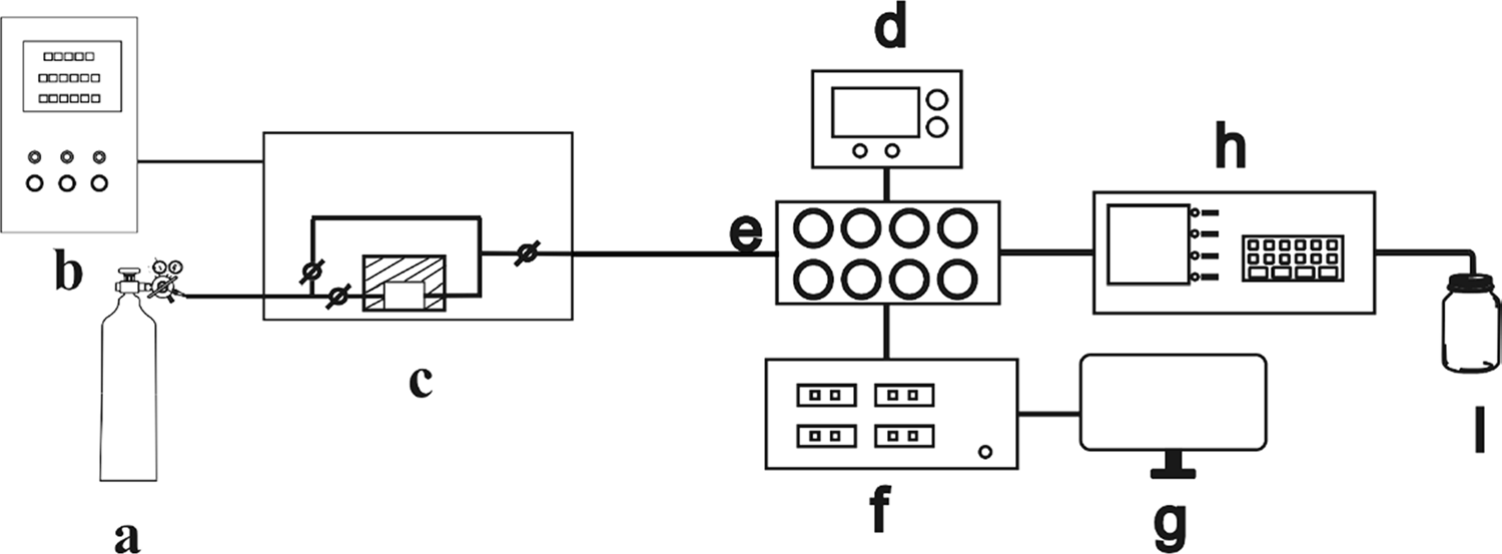
Dynamic testing platform: **a** Gas cylinder; **b** Generation system controller; **c** Dynamic generation system; **d** Direct-current potential regulator; **e** Eight-channel testing chamber; **f** Test source table; **g** Workstation; **h** Concentration monitor; **i** Exhaust cylinder [17]

The gas sensor was evaluated as follows. First, the as-prepared gas-sensing device was aged for ≥ 72 h using an aging platform. The device was then placed in an eight-channel testing chamber (Fig. 2e), and the chemical toxicants or simulants were placed as shown in Fig. 2c. The concentration of the target gas could be adjusted by controlling the temperature and gas flow rate in the generation chamber using a controller (Fig. 2b). The working temperature of the device could be adjusted by controlling the temperature of the heating wire using a power source (Fig. 2d). The real-time concentration data of the generation chamber were detected using a monitor (Fig. 2h). The real-time resistance data shown in Fig. 2f could be obtained using the workstation shown in Fig. 2g for further data processing.

The sensitivity of the sensor is defined as $S={R}_{a}/{R}_{g}$, where ${R}_{a}$ and ${R}_{g}$ are the respective resistances of the device in air and the stable state under the testing gas. The response (${t}_{res}$) and recovery times (${t}_{rec}$) of the device are defined as the times required for the gas-sensitive component to reach 90% of the total changes in resistance during adsorption and desorption.

## Results and discussion

### Structure and morphology

**Fig. 3:**
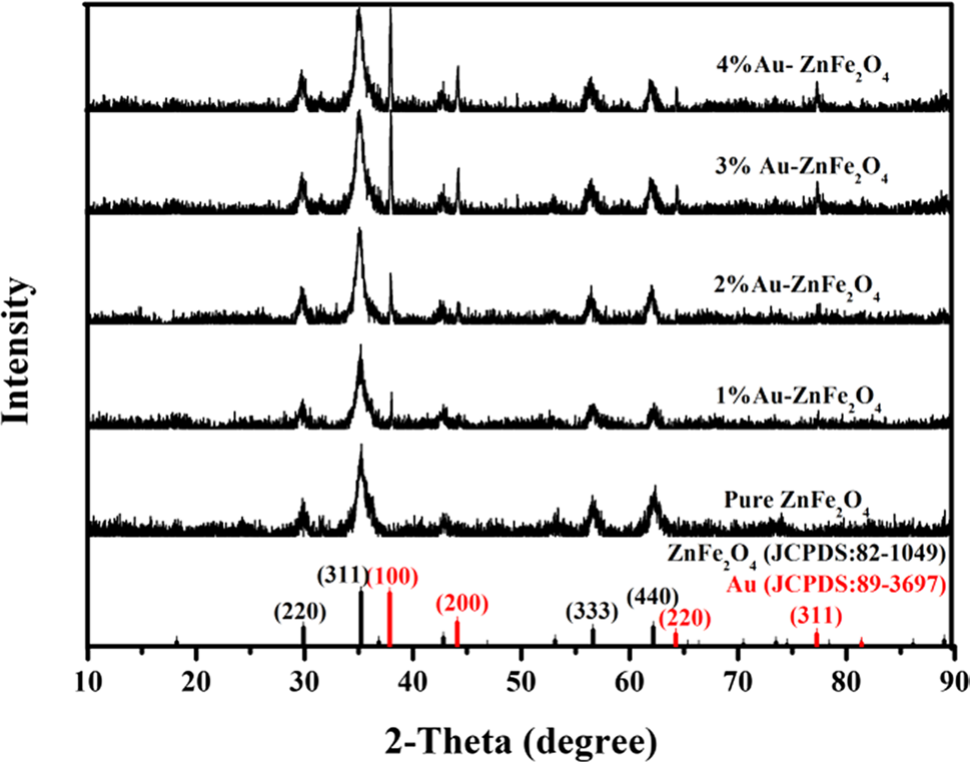
XRD patterns of the Au-loaded ZnFe_2_O_4_ samples with different Au loading ratios

Figure 3 shows the XRD patterns of the as-prepared Au-loaded ZnFe_2_O_4_ samples. The diffraction peaks of the samples with five different Au loading ratios at 2θ = 29.92°, 35.24°, 56.61°, and 62.16° are attributed to the (220), (311), (333), and (440) crystal planes of ZnFe_2_O_4_, respectively, based on the standard diffraction pattern (JCPDS: 82–1049) [29]. Conversely, the diffraction peaks at 2θ = 38.18°, 44.37°, 64.56°, and 77.54° may be attributed to the (100), (200), (220), and (311) crystal planes of Au, respectively, according to the standard diffraction pattern (JCPDS: 89–3697) [34]. Moreover, the intensities of the Au diffraction peaks increase with increasing Au loading. Notably, no peaks representing impurities or other crystalline phases are observed in the XRD patterns. Therefore, the five as-synthesized samples, including pure ZnFe_2_O_4_ and ZnFe_2_O_4_ loaded with small amounts of Au, exhibit high purities and no other heterogeneous phases.

**Fig. 4:**
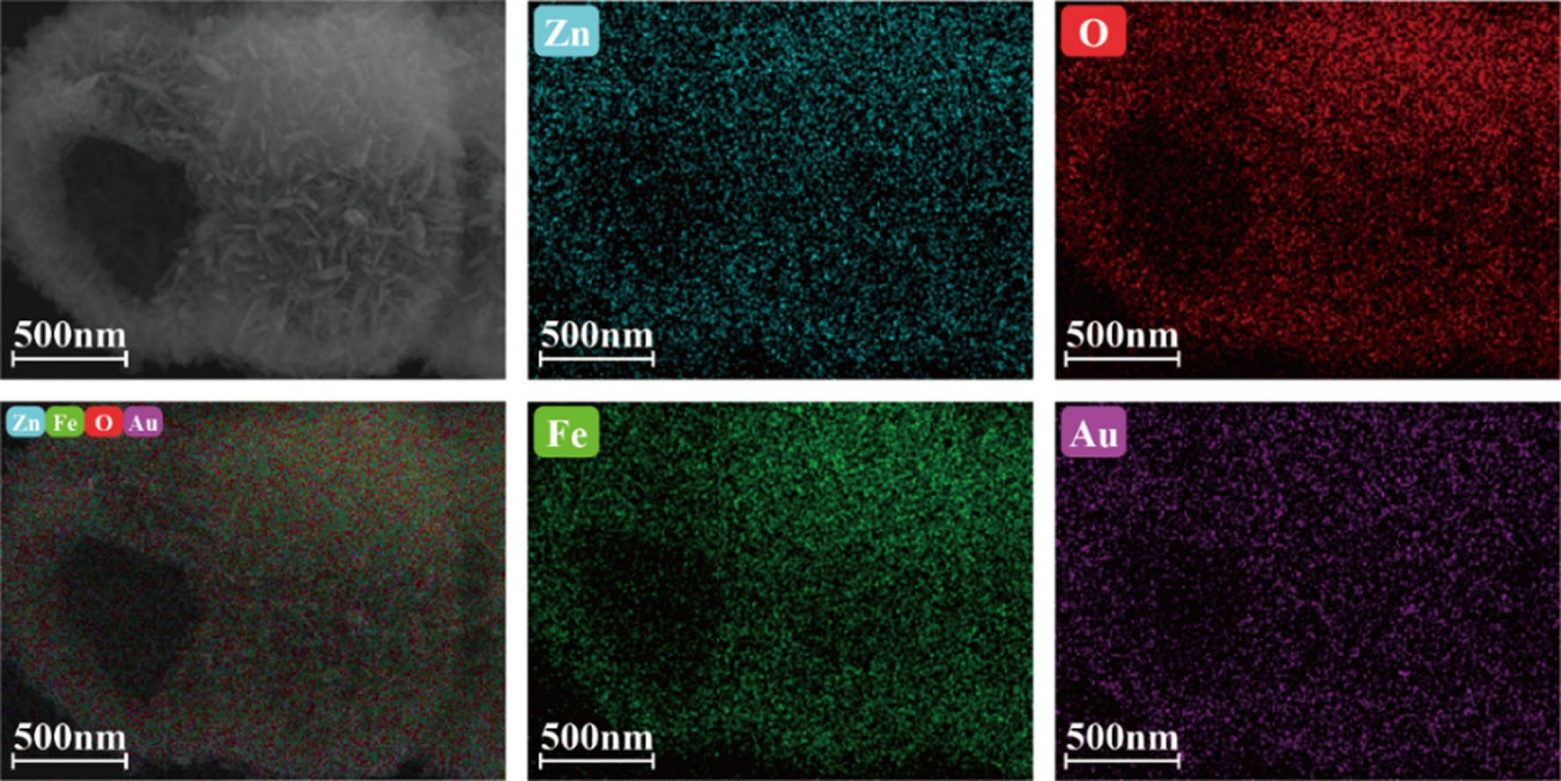
EDS maps of 2 wt.% Au-loaded ZnFe_2_O_4_

EDS of the as-prepared ZnFe_2_O_4_ samples was performed, and the corresponding mass fractions of each element are listed in Table 1. The mass fractions of Au in these five samples are 0, 0.87, 2.13, 2.86, and 4.22%, which are close to the theoretical values. Therefore, the method of loading Au onto the ZnFe_2_O_4_ samples is viable. Based on the EDS results for 2 wt.% Au-ZnFe_2_O_4_ (Fig. 4), the presence of Zn, Fe, O, and Au in the sample and their uniform distributions within the ZnFe_2_O_4_ hollow microspheres are consistent with the XRD results.

**Table 1:** Mass fractions of the elements in the Au-loaded ZnFe_2_O_4_ samples with different Au loading ratios

Material	Fe (%)	Zn (%)	O (%)	Au (%)
Pure-ZnFe_2_O_4_	45.67	26.41	27.92	0
1 wt% Au—ZnFe_2_O_4_	46.82	25.73	26.58	0.87
2 wt% Au—ZnFe_2_O_4_	43.65	24.81	29.41	2.13
3 wt% Au—ZnFe_2_O_4_	44.86	25.27	27.01	2.86
4 wt% Au—ZnFe_2_O_4_	45.24	23.93	26.61	4.22

**Fig. 5:**
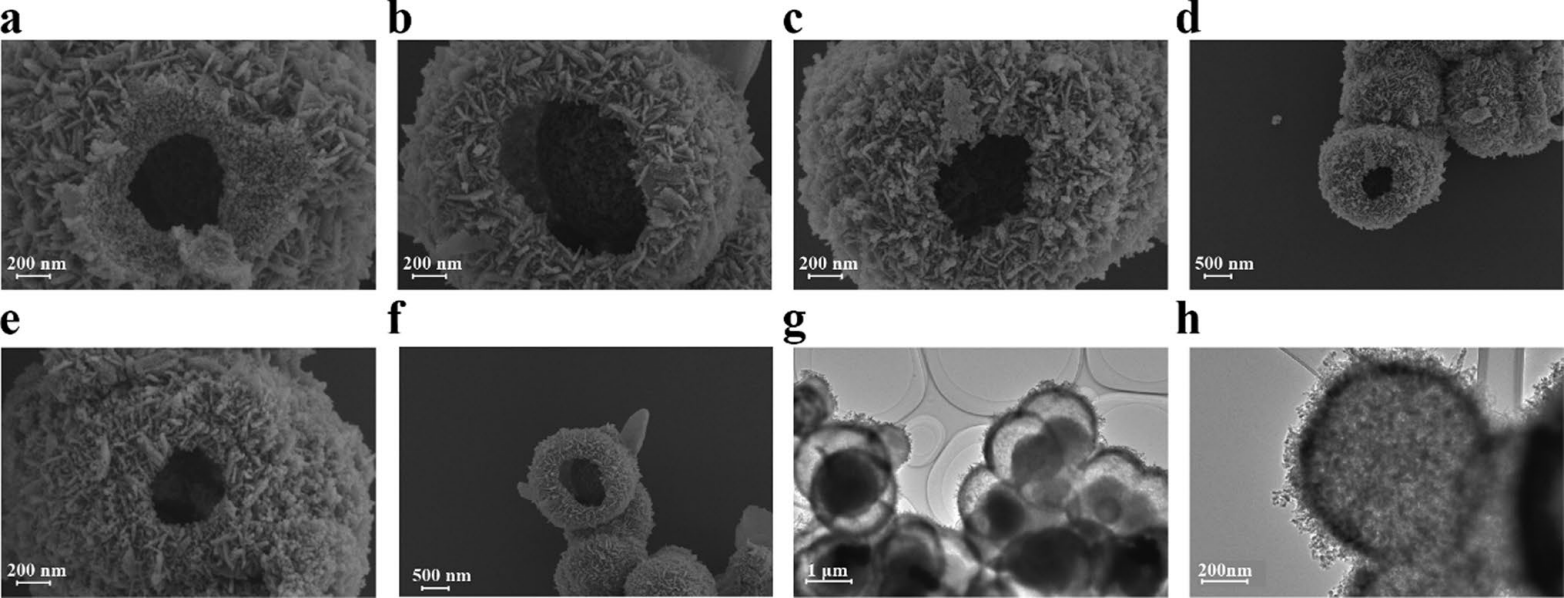
SEM and TEM images of the Au-loaded ZnFe_2_O_4_ samples: **a**–**f** SEM images of pure ZnFe_2_O_4_, 1 wt.% Au-ZnFe_2_O_4_, 2 wt.% Au-ZnFe_2_O_4_, 3 wt.% Au-ZnFe_2_O_4_, and 4 wt.% Au-ZnFe_2_O_4_; **g**–**h** TEM images of 2 wt.% Au-ZnFe_2_O_4_

According to the SEM images of the ZnFe_2_O_4_ samples loaded with Au at various ratios (Fig. 5a–f), the as-prepared samples exhibit hollow microsphere morphologies with rough surfaces. In addition, clear gaps are observed between the nanosheets on the microsphere surfaces, indicating their porous and loose characteristics. This porous structure may effectively adsorb gas molecules and facilitate their diffusion, leading to excellent gas-sensing performance. As the level of Au doping increases, no significant changes in the sizes or overall hollow structures of the microspheres are observed. Figure 5g, h show the TEM images of 2 wt.% Au-ZnFe_2_O_4_. The sharp contrast between the dark edges and white gaps in the image further confirms the morphology as a surface-roughened hollow microsphere structure, with a corresponding diameter of approximately 1.2 µm.

**Fig. 6:**
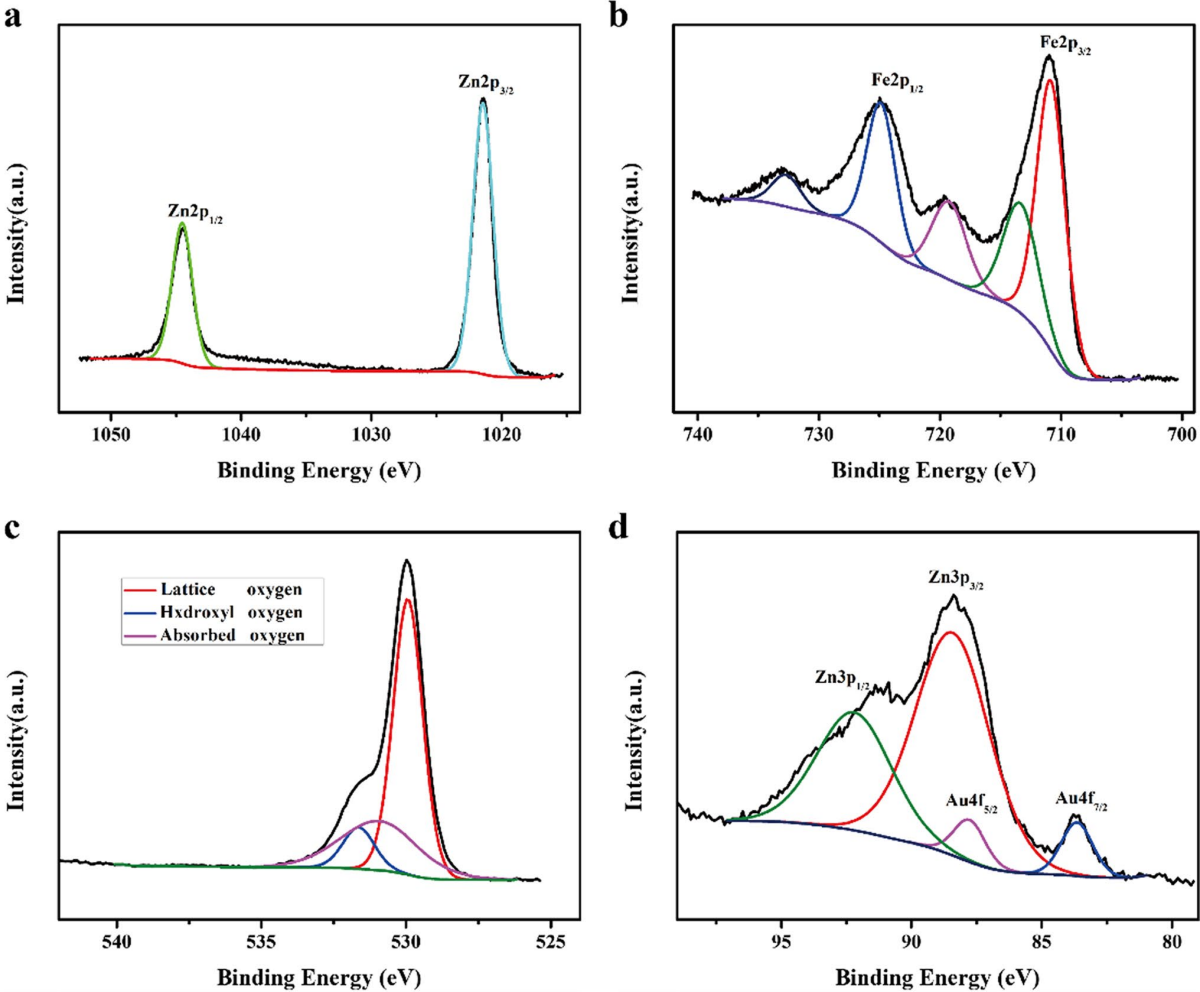
XPS profiles of 2 wt.% Au-loaded ZnFe_2_O_4_: **a** Zn 2p; **b** Fe 2p; **c** O 1s; and **d** Au 4f

XPS was performed to investigate the surface chemical compositions and valence states of the 2 wt.% Au-loaded ZnFe_2_O_4_ hollow microsphere samples; the results are shown in Fig. 6. In Fig. 6a, the two Zn 2p peaks at 1044.53 and 1021.46 eV are assigned to Zn 2p_1/2_ and Zn 2p_3/2_, respectively, indicating that the valence state of Zn in the sample is + 2 [50]. In Fig. 6b, the peaks at 713.33 and 710.83 eV may be ascribed to Fe 2p_3/2_ at the tetrahedral (A-site) and octahedral (B-site) sites of the ZnFe_2_O_4_ spinel structure, respectively. The peak at 724.86 eV may be attributed to Fe 2p_1/2_, whereas the peaks at 732.69 and 719.24 eV represent the satellite peaks of Fe 2p, indicating that the valence state of Fe in the sample is + 3 [29]. In Fig. 6c, the O 1s spectrum displays three peaks at 532.72, 530.92, and 529.93 eV, corresponding to the hydroxyl, surface-adsorbed, and lattice oxygen in the sample, respectively [28]. Based on XPS peak area measurements, the respective relative proportions of these oxygen species are 9.8, 28.8, and 61.4%, and surface-adsorbed oxygen acts favorably to improve the gas-sensing performance of sensitive materials [51]. Based on the Au 4f spectrum in Fig. 6d, the peaks at 92.21 and 88.42 eV are respectively assigned to Zn 3p_1/2_ and Zn 3p_3/2_ of Zn^2+^. Additionally, the peaks at 87.83 and 83.62 eV represent Au 4f_5/2_ and Au 4f_7/2_, respectively, and thus, the valence state of Au in the sample is + 1 [52]. The results of XPS, as shown in Fig. 6, are consistent with those of XRD and EDS, further confirming the successful preparation of the Au-loaded ZnFe_2_O_4_ hollow microspheres.

### Gas-sensing properties

**Fig. 7:**
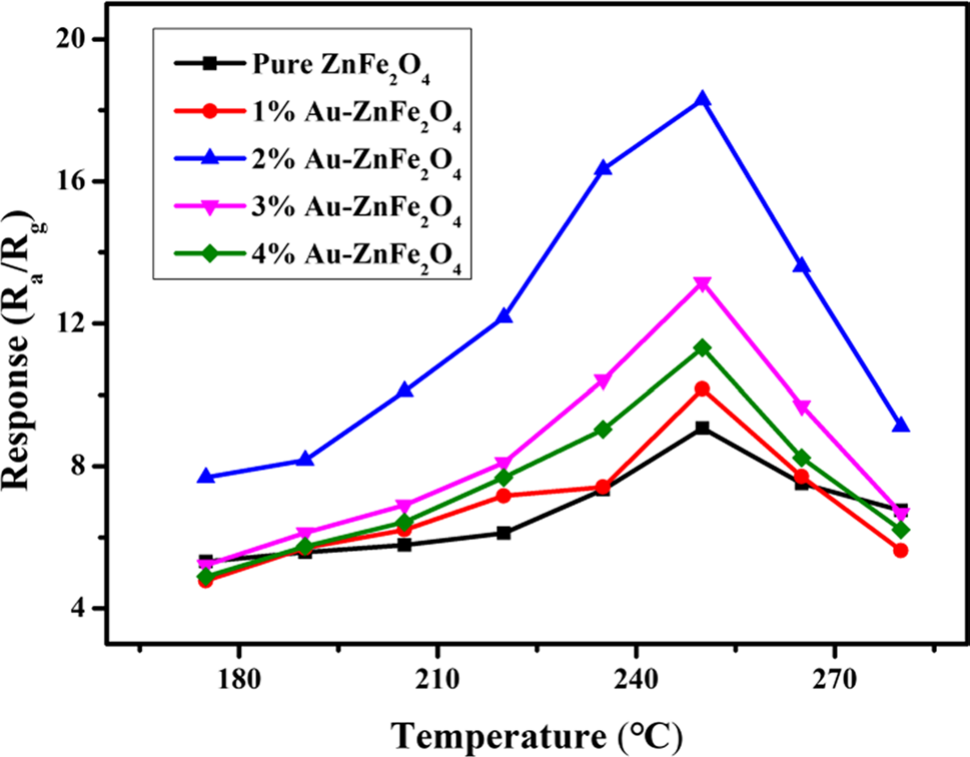
Response curves of the ZnFe_2_O_4_ sensors with different Au loading ratios toward 1 ppm of 2-CEES at various working temperatures

**Fig. 8:**
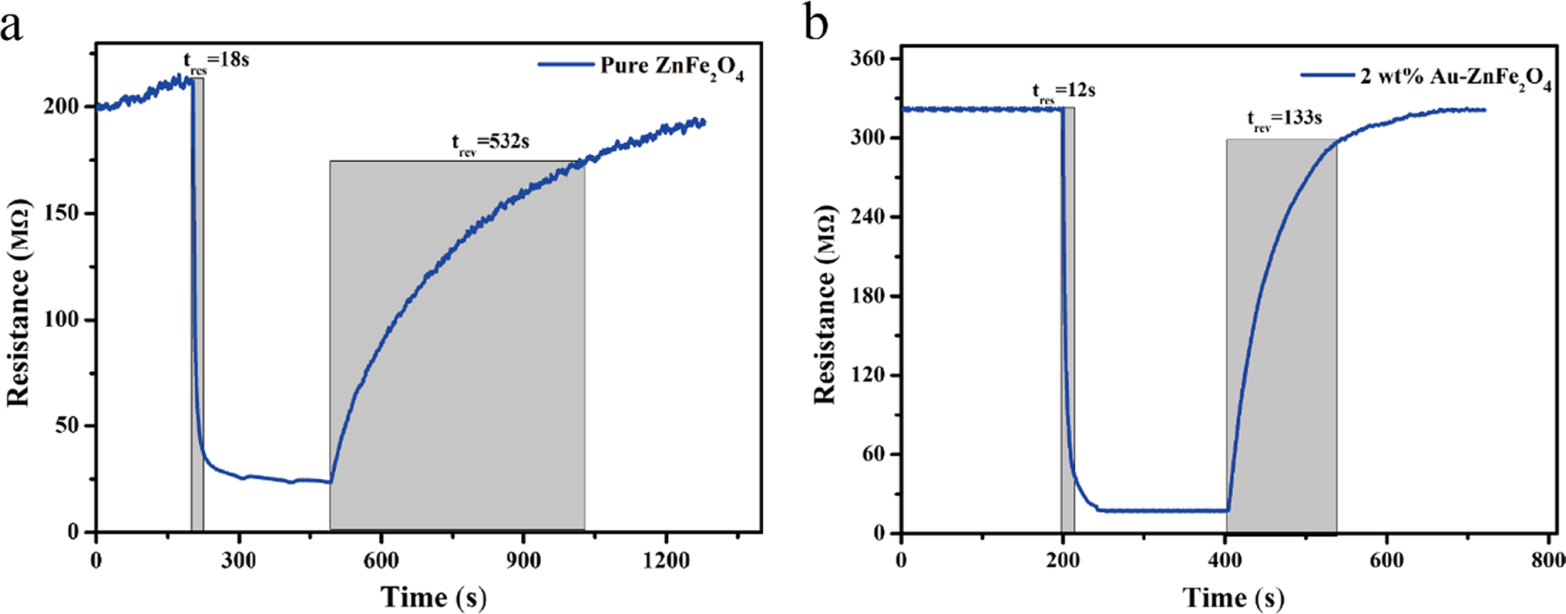
Response and recovery curves: Sensors based on **a** pure ZnFe_2_O_4_ and **b** 2 wt.% Au-ZnFe_2_O_4_ at 250 °C under 1 ppm of 2-CEES

The performance of a gas sensor strongly depends on its operating temperature. Figure 7 shows the evaluated sensitivities to 1 ppm of 2-CEES for gas sensors based on Au-loaded ZnFe_2_O_4_ hollow microspheres with different Au loading ratios, operating within the temperature range of 175–280 °C. The responses of the gas sensors to 2-CEES increase and then decrease with an increase in the working temperature, indicating the presence of optimal operating temperatures. This may be explained by the enhancement in target gas diffusion and the increase in the amount of surface-adhered O^–^ on the surface of the sensitive material as the temperature is gradually increased, leading to improved sensitivity. However, as the temperature continues to increase, the diffusion of the target gas gradually becomes saturated. Consequently, the desorption of the target gas plays a dominant role, thereby reducing the sensitivity [26]. In this study, the optimal working temperature of the as-prepared gas sensors is 250 °C. Based on the gas-sensing performances of the Au-loaded ZnFe_2_O_4_ hollow microspheres with different Au loading ratios, the sensor performance initially improves and then deteriorates with increasing Au loading. The gas sensor based on the 2 wt.% Au-loaded ZnFe_2_O_4_ hollow microspheres exhibits the highest sensitivity, and Fig. 8 shows its response and recovery curves. At the optimal working temperature of 250 °C, the gas sensor based on the 2 wt.% Au-loaded ZnFe_2_O_4_ hollow microspheres reaches a sensitivity toward 1 ppm of 2-CEES of 18.29, which is approximately double that of the undoped sensor (9.07). The corresponding response/recovery times also decrease from 18/546 to 12/133 s, respectively, suggesting that Au loading on ZnFe_2_O_4_ may improve the gas-sensing performance. This improvement is mainly attributed to the effect of electronic and chemical sensitization by Au loading [53].

**Fig. 9:**
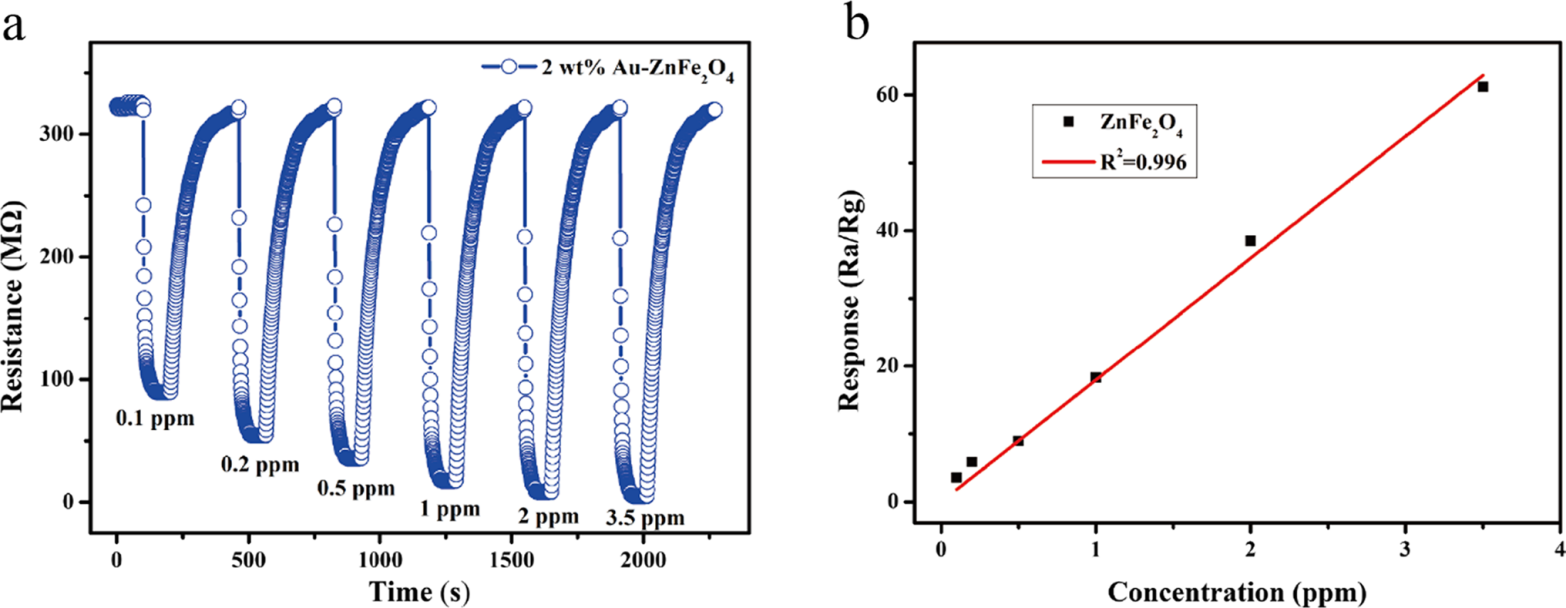
Response sensitivity of the sensor based on the 2 wt.% Au-loaded ZnFe_2_O_4_ hollow microspheres toward 2-CEES within the concentration range of 0.1–3.5 ppm at the optimal working temperature of 250 °C: **a** Response-recovery curves and **b** linear fitting plot

Figure 9a shows the sensing performance of the gas sensor based on the 2 wt.% Au-loaded ZnFe_2_O_4_ hollow microspheres toward 2-CEES within the concentration range of 0.1–3.5 ppm at the optimal working temperature of 250 °C. With an increase in the concentration of 2-CEES, the sensor’s sensitivity is enhanced. The sensitivity may still reach 3.56 at a 2-CEES concentration as low as 0.1 ppm, suggesting that the detection limit of the as-prepared sensor is < 0.1 ppm. As shown in Fig. 9b, the sensor exhibits good linearity within the 2-CEES concentration range of 0.1–3.5 ppm, and the linear correlation coefficient is 0.996, which is highly favorable for the quantitative application of the sensor.

**Fig. 10:**
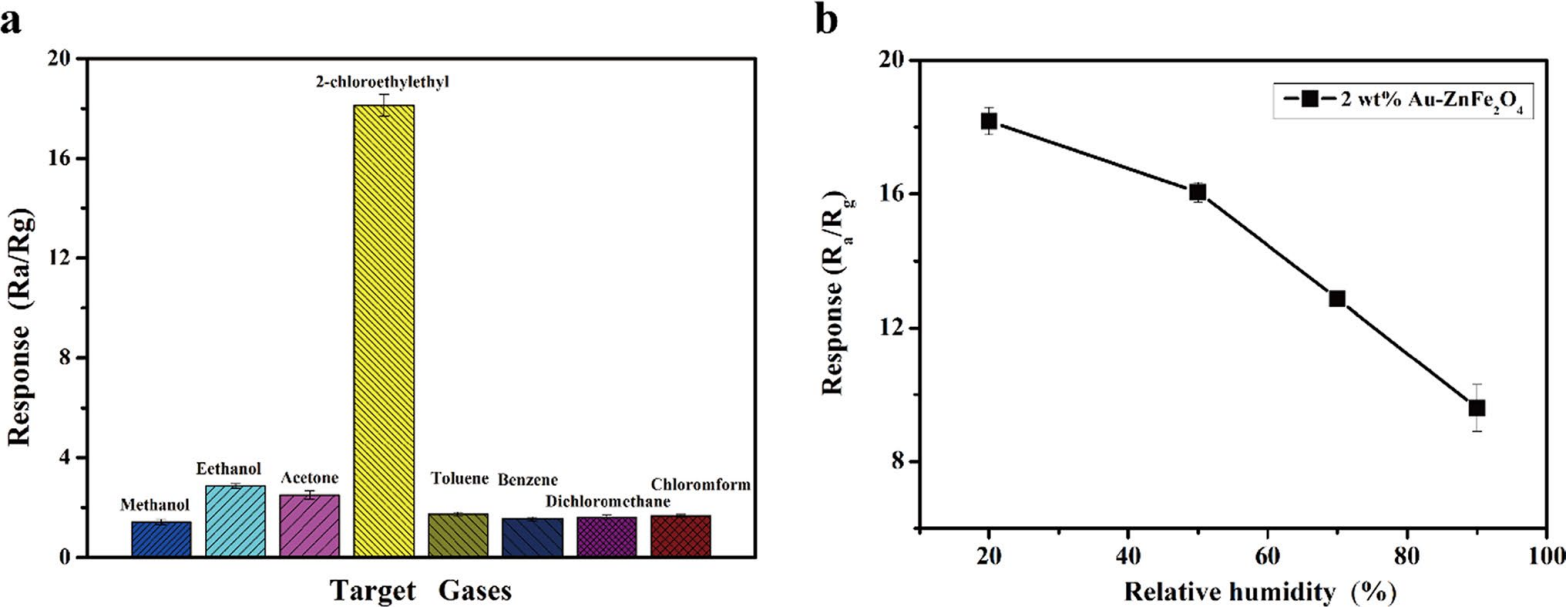
**a** Responses of the 2 wt.% Au-loaded ZnFe_2_O_4_ hollow microsphere-based sensor to 1 ppm of different gases at 250 °C. **b** Responses of the 2 wt.% Au-loaded ZnFe_2_O_4_ hollow microsphere-based sensor to 1 ppm of 2-CEES at different humidities

To verify the selectivity of the 2 wt.% Au-loaded ZnFe_2_O_4_ hollow microsphere-based gas sensor, it was evaluated for its sensitivities toward 1 ppm of seven different volatile organic gases at the optimal working temperature of 250 °C (Fig. 10a). The sensor exhibits the highest sensitivity towards 2-CEES (18.29) compared to those towards methanol (1.52), ethanol (2.96), acetone (2.64), toluene (1.83), benzene (1.62), dichloromethane (1.71), and trichloromethane (1.73), indicating the good selectivity of the as-prepared sensor based on the 2 wt.% Au-loaded ZnFe_2_O_4_ hollow microspheres towards 2-CEES. At the operating temperature of 250 ℃, the gas sensitivities toward 1 ppm of 2-CEES at humidity levels ranging from 20 to 90% were evaluated. As shown in Fig. 10b, the sensitivity of the sensor decreases with increasing humidity. The sensitivity is reduced by approximately 50% at a humidity of 90%, which may be attributed to the competitive adsorption of H_2_O vapor [23].

**Fig. 11:**
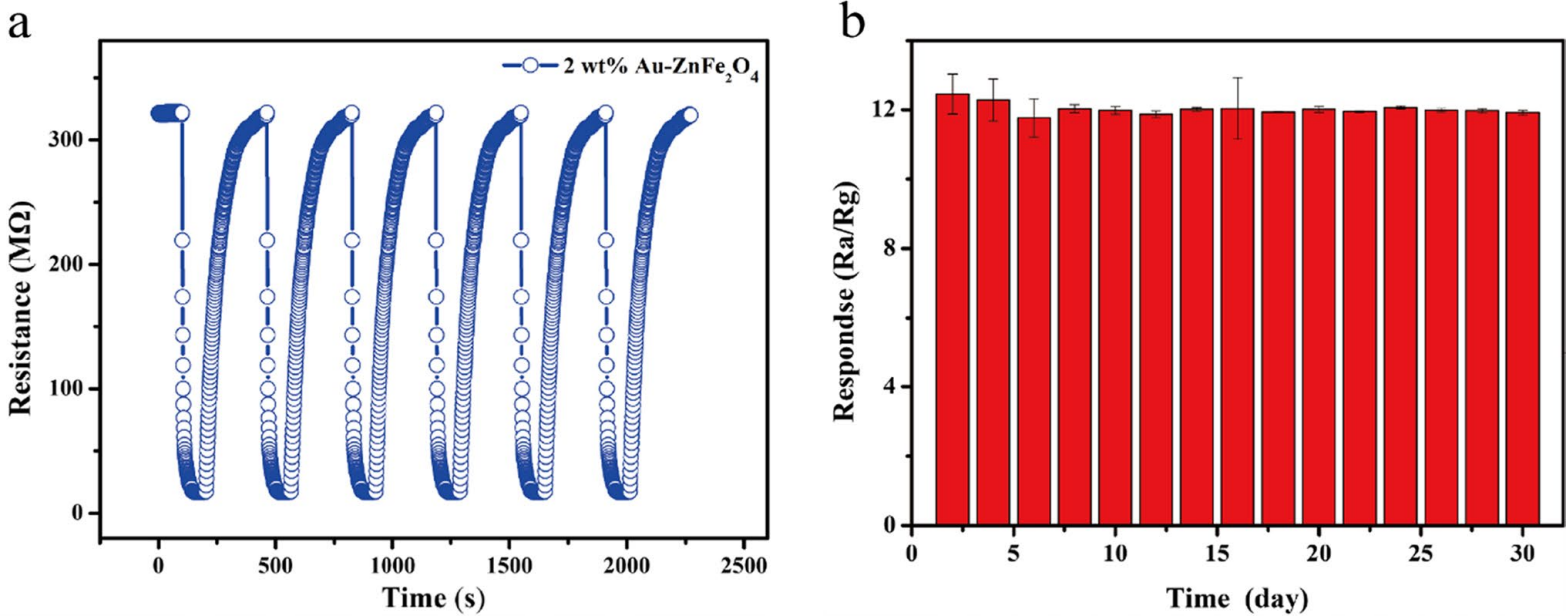
**a** Response and recovery curves of the sensor at 250 °C under 1 ppm of 2-CEES in the repeatability study. **b** Sensitivity of the sensor over 30 d of continuous use

At a working temperature of 250 °C, continuous response and recovery studies were performed using 1 ppm of 2-CEES. As shown in Fig. 11a, the response and recovery curves are consistent, indicating that the sensor exhibits good repeatability. Additionally, the sensitivity of the sensor was monitored over 30 d. As depicted in Fig. 11b, the sensitivity remains relatively stable over the entire period, indicating the good long-term stability and suitability of the sensor for use in continuous monitoring applications.

### Mechanistic analysis

**Fig. 12:**
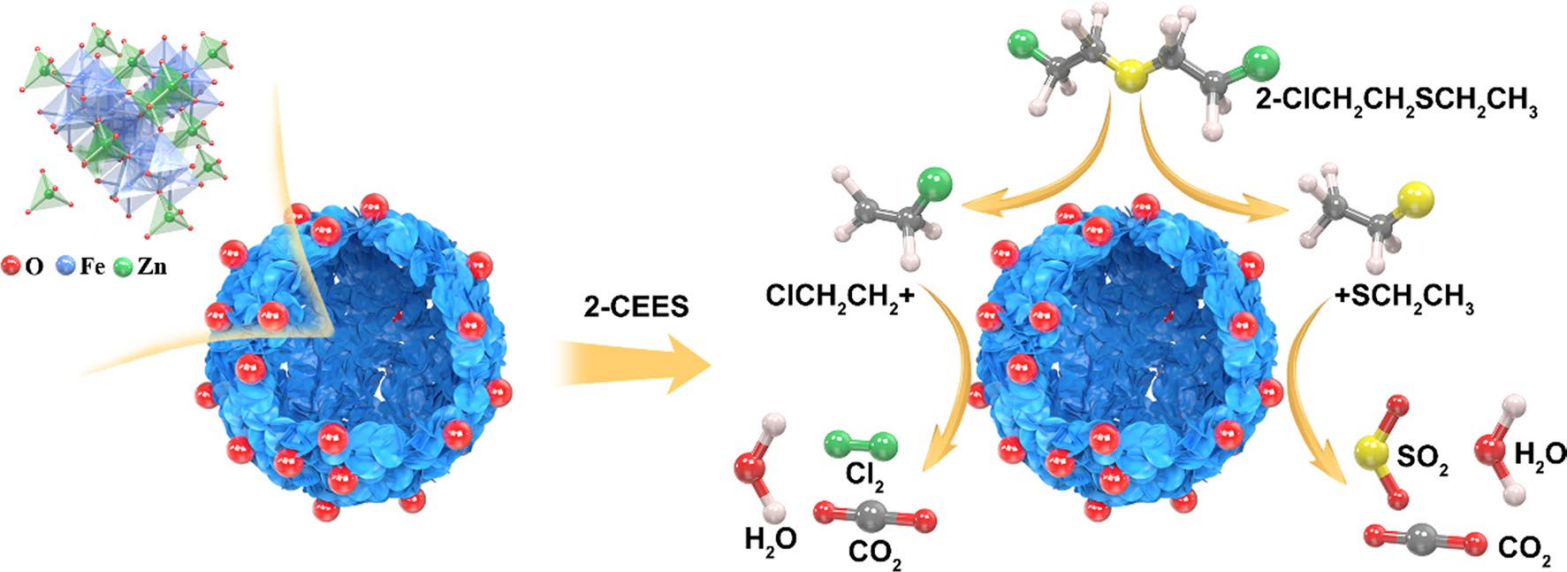
Gas-sensing mechanism of the ZnFe_2_O_4_ hollow microsphere-based gas sensor for 2-CEES

ZnFe_2_O_4_ is a typical n-type semiconductor material. Therefore, the gas-sensing mechanism of the ZnFe_2_O_4_-based gas sensor for 2-CEES is primarily attributed to the interactions between 2-CEES and the surface-adsorbed oxygen of the ZnFe_2_O_4_ sensing material. These interactions result in a change in the surface electrical resistance of the material, and the corresponding gas-sensing mechanism is illustrated in Fig. 12.

In ambient air, O_2_ molecules are adsorbed on the surface of the sensing material and electrons may be extracted from the conduction band, resulting in the formation of more active adsorbed O species (O_2_^–^, O^–^ and O^2–^) [54, 55]. This may result in the formation of an electron-depleted layer on the surface of the material, thereby enhancing its electrical resistance. At temperatures of < 150 °C, the adsorbed O mainly occurs as O_2_^–^, as previously reported [56], and the adsorbed O mainly occurs as O^–^ in the temperature range of 150–400 °C. At a temperature of > 400 °C, the adsorbed O mainly occurs as O^2–^, and the reaction is expressed as follows:

1 $${{\text{O}}}_{2}\left({\text{gas}}\right)\to {{\text{O}}}_{2}\left({\text{ads}}\right)$$

2 $${{\text{O}}}_{2}\left({\text{ads}}\right)+{{\text{e}}}^{-}\to {{\text{O}}}_{2}^{-}\left({\text{ads}}\right)$$

3 $${{\text{O}}}_{2}^{-}\left({\text{ads}}\right)+{{\text{e}}}^{-}\to 2{{\text{O}}}^{-}\left({\text{ads}}\right)$$

4 $${{\text{O}}}^{-}\left({\text{ads}}\right)+{{\text{e}}}^{-}\to {{\text{O}}}^{2-}\left({\text{ads}}\right)$$

When the ZnFe_2_O_4_ sensor is exposed to the 2-CEES atmosphere, 2-CEES is initially decomposed into two free radicals [17], i.e., ${{\text{ClCH}}}_{2}{{\text{CH}}}_{2}^{\bullet }$ and ${}^{\bullet }{{\text{SCH}}}_{2}{{\text{CH}}}_{3}$, which are adsorbed at Lewis acid sites via the Cl and S groups, leading to the surface adsorption of the material. These free radicals may interact with the O^–^ adsorbed on the material surface, following which the electrons captured by O^–^ may be released back to the conduction band. The thickness of the electron-depleted layer on the material surface decreases, resulting in a reduction in electrical resistance. This reaction can be expressed as follows:

5 $$2{\text{CEES}}\to {{\text{ClCH}}}_{2}{{\text{CH}}}_{2}^{\bullet }+{}_{ }{}^{\bullet }{{\text{SCH}}}_{2}{{\text{CH}}}_{3}$$

6 $$2{{\text{ClCH}}}_{2}{{\text{CH}}}_{2}+8{{\text{O}}}^{-}\to 2{{\text{CO}}}_{2}+{{\text{Cl}}}_{2}+4{{\text{H}}}_{2}{\text{O}}+8{{\text{e}}}^{-}$$

7 $$2{{\text{CH}}}_{3}{{\text{CH}}}_{2}{\text{S}}+13{{\text{O}}}^{-}\to 2{{\text{SO}}}_{2}+5{{\text{H}}}_{2}{\text{O}}+2{{\text{CO}}}_{2}+13{{\text{e}}}^{-}$$

The high gas sensitivity response of the Au-loaded ZnFe_2_O_4_ microsphere-based gas sensor to 2-CEES can be attributed to the following four points: First, due to the difference between Zn^2+^and Fe^3+^ (such as ionic radius, electronegativity, etc.), ZnFe_2_O_4_ with a narrow bandgap width (about 2.1 eV) is more susceptible to heat excitation and produces more electrons, and the oxygen in the air is also easier to capture electrons from the conduction band, producing more adsorbed oxygen, thereby improving the sensitivity. Second, the hollow structure of the ZnFe_2_O_4_ microspheres composed of nanosheets is rough and porous, which can provide more active sites and gas adsorption/desorption channels. Again, the Au-modified ZnFe_2_O_4_ material has the ability to adsorb and crack oxygen more efficiently, so that the width of the depletion layer on the surface of the material increases, resulting in a decrease in the conductivity of the sensitive material, and as can be seen from Fig. 8, the matrix resistance of the 2 wt% ZnFe_2_O_4_ material is significantly higher than that of the undoped ZnFe2O4. In addition, Au can be used as a catalyst to cleave part of 2-CEES into many more reactive groups, which are more likely to react with oxygen adsorbed on the surface of the material, thereby catalyzing chemical reactions between gases. That is, the electron sensitization and chemical sensitization effects of precious metals, therefore, their response value and response recovery speed have been greatly improved. Finally, based on the high affinity of Au–S, it may be the main reason for its increased selectivity for 2-CEES.

## Conclusions

Au-loaded ZnFe_2_O_4_ hollow microspheres with different Au loading ratios were successfully synthesized through a solvothermal method. The chemical compositions and morphological characteristics were analyzed using XRD, EDS, SEM, TEM, and XPS. The sensor based on the as-prepared 2wt% Au-loaded ZnFe_2_O_4_ microspheres displayed excellent gas-sensing performance towards the mustard gas simulant 2-CEES. At the optimal temperature of 250 °C, the sensor exhibited a high sensitivity of 18.29 toward 1 ppm of 2-CEES, and the respective response and recovery times were 12 and 133 s. Additionally, the sensor displayed a sensitivity of 3.56 toward concentrations of 2-CEES as low as 0.1 ppm and good selectivity in the presence of methanol, ethanol, acetone, benzene, toluene, dichloromethane, and trichloromethane. The sensor exhibited good repeatability over multiple consecutive response and recovery cycles, and minimal sensitivity fluctuations were observed over a continuous monitoring period of 30 d, indicating that the sensor demonstrated excellent long-term stability. The sensor also exhibited good linearity in the 2-CEES concentration range of 0.1–3.5 ppm. The Au-loaded ZnFe_2_O_4_-based sensor has considerable potential for detecting toxic chemical agents and their simulants.

## Data Availability

Data available on request from the authors.
